# The Digestive System of the Two-Spotted Spider Mite, *Tetranychus urticae* Koch, in the Context of the Mite-Plant Interaction

**DOI:** 10.3389/fpls.2018.01206

**Published:** 2018-09-11

**Authors:** Nicolas Bensoussan, Vladimir Zhurov, Sota Yamakawa, Caroline H. O'Neil, Takeshi Suzuki, Miodrag Grbić, Vojislava Grbić

**Affiliations:** ^1^Department of Biology, The University of Western Ontario, London, ON, Canada; ^2^Graduate School of Bio-Applications and Systems Engineering, Tokyo University of Agriculture and Technology, Tokyo, Japan; ^3^Robarts Research Institute, The University of Western Ontario, London, ON, Canada

**Keywords:** gut, digestion, detoxification, plant-pest interaction, pest, histology

## Abstract

The two-spotted spider mite (TSSM), *Tetranychus urticae* Koch (Acari: Tetranychidae), is one of the most polyphagous herbivores, feeding on more than 1,100 plant species. Its wide host range suggests that TSSM has an extraordinary ability to modulate its digestive and xenobiotic physiology. The analysis of the TSSM genome revealed the expansion of gene families that encode proteins involved in digestion and detoxification, many of which were associated with mite responses to host shifts. The majority of plant defense compounds that directly impact mite fitness are ingested. They interface mite compounds aimed at counteracting their effect in the gut. Despite several detailed ultrastructural studies, our knowledge of the TSSM digestive tract that is needed to support the functional analysis of digestive and detoxification physiology is lacking. Here, using a variety of histological and microscopy techniques, and a diversity of tracer dyes, we describe the organization and properties of the TSSM alimentary system. We define the cellular nature of floating vesicles in the midgut lumen that are proposed to be the site of intracellular digestion of plant macromolecules. In addition, by following the TSSM's ability to intake compounds of defined sizes, we determine a cut off size for the ingestible particles. Moreover, we demonstrate the existence of a distinct filtering function between midgut compartments which enables separation of molecules by size. Furthermore, we broadly define the spatial distribution of the expression domains of genes involved in digestion and detoxification. Finally, we discuss the relative simplicity of the spider mite digestive system in the context of mite's digestive and xenobiotic physiology, and consequences it has on the effectiveness of plant defenses.

## Introduction

Plant-pest interactions represent an elaborate interplay between organisms whose complexity reflects a constant evolutionary arms-race between a plant and a guild of its pests. Plants evolved a myriad of strategies to deter herbivory, ranging from the manipulation of multitrophic interactions through the recruitment of pest natural enemies, to the placement of physical or chemical barriers that repel herbivores and prevent consumption of plant tissues. In addition, as herbivores feed on plants to acquire nutrients, they also ingest a wide range of plant defense compounds that interfere with essential herbivore physiological processes (Howe and Jander, 2008). The exposure to plant-imposed barriers has been an important driver of herbivore evolution for millions of years, resulting in the development of counter mechanisms that enable herbivores to overcome plant defenses (Heidel-Fischer and Vogel, 2015). As the alimentary tract is one of the major sites where plant-herbivore warfare takes place, knowledge of its cellular organization and physiology is essential for the functional understanding of plant-herbivore interactions.

Insect herbivores have been excellent models for understanding the interplay between plant- and insect-derived molecules acting in the gut. Identification of plant toxic metabolites and their detoxification counterparts have been described for several plant-pest interactions. For example, allelic variation, gene duplication, overexpression and the subfunctionalization of cytochrome P450 proteins enabled the parsnip webworm (*Depressaria pastinacella*) and the black swallowtail (*Papilio polyxenes*) to feed on furanocoumarin-containing plants (Mao et al., 2006, 2007; Wen et al., 2006), and the aphid *Myzus persicae* to utilize nicotine-accumulating tobacco as a host (Bass et al., 2013). In addition to compounds that act as xenobiotics, plants synthesize defensive proteins that directly target gut physiology. Lectins, chitin-binding proteins, chitinases and some proteases disrupt the peritrophic matrix (Zhu-Salzman et al., 1998; Vandenborre et al., 2011), a structure that lines luminal surface of the alimentary canal and aids in digestion and protection of the midgut epithelial cells. In addition, plants synthesize enzymes like proteinase inhibitors (PIs) that interfere with the digestion of plant nutrients, and amino acid-degrading enzymes (e.g., arginase (ARG2) and threonine deaminase (TD2) (Chen et al., 2005; Gonzales-Vigil et al., 2011), which reduce the availability of nutrients that herbivores require for development. The knowledge of gut physiology and its cellular organization is of primary importance for understanding the interactions between plant defense molecules and the cellular environment of insect guts. For example, gut pH profoundly affects the effectiveness of ARG2 and TD2. As these enzymes are most active within an alkaline pH, they are effective in restricting herbivory of insects with alkaline guts, such as lepidopteran larvae (Gu et al., 1999; Chen et al., 2004, 2007; Fowler et al., 2009; Chung and Felton, 2011; Gonzales-Vigil et al., 2011), but are ineffective against herbivores with acidic guts, like the Colorado potato beetle (Felton et al., 1992; Gonzales-Vigil et al., 2011). Recent advances in DNA and RNA sequencing technologies further enabled mapping of the expression of genes to different gut domains, thereby correlating the anatomical and genomic features in some insect herbivores (Neira Oviedo et al., 2008; Chung et al., 2009).

The two-spotted spider mite (TSSM), *Tetranychus urticae* (Koch), is a chelicerate herbivore with an exceptionally wide host range (Migeon et al., 2010). The TSSM's extreme polyphagy indicates that TSSM has an outstanding ability to adapt its digestive physiology and to overcome a wide range of defenses imposed by different host plants (Rioja et al., 2017). TSSM's xenobiotic responsiveness, an ability to detoxify many diverse phytochemicals, is also associated with its ability to readily develop resistance to pesticides (Dermauw et al., 2018). Characterization of the TSSM feeding pattern showed that TSSMs consume the content of single mesophyll cell at a time (Bensoussan et al., 2016). As TSSMs ingest plant cellular content, they also take up mesophyll parenchyma-localized defense compounds, suggesting that both nutrient digestion and detoxification of plant defense compounds occur in digestive tract.

**Figure 1:**
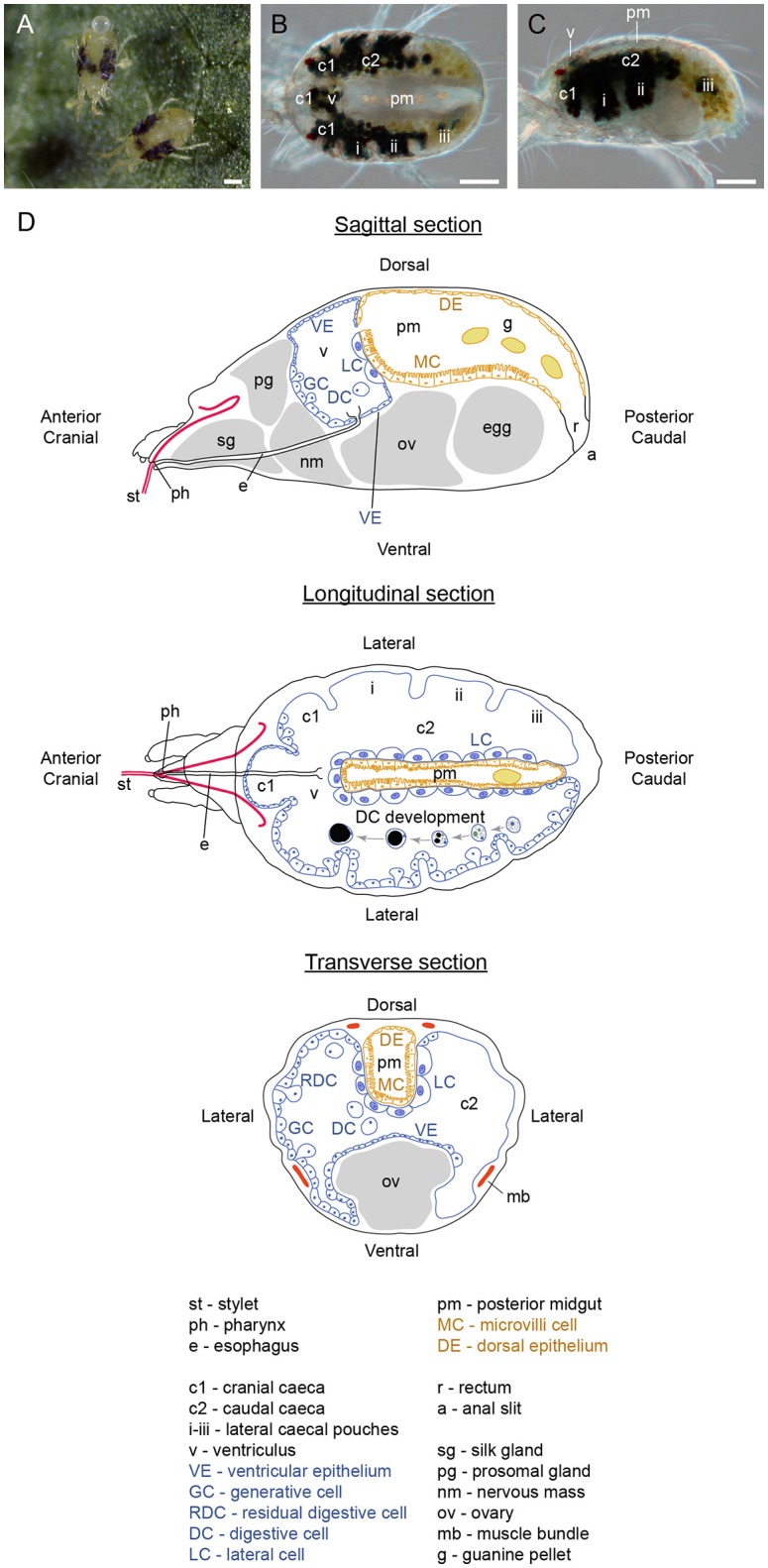
Organization of spider mite body. **(A)** Spider mite females on bean (*Phaseolus vulgaris*) leaf. **(B)** Dorsal view of female spider mite. **(C)** Lateral view of female spider mite. **(D)** Schematics of spider mite internal anatomy. Scale bars **(A–C)**: 100 μm.

The two-spotted spider mite was named for its two distinct dark spots that originate from the internal gut content visible through the semitransparent cuticle (Figures 1A–C). TSSMs, like insects, have a complete digestive system that consists of a foregut, midgut and hindgut. However, TSSM digestive physiology deviates from a characteristic insect digestive system. For example, insects digest nutrients extracellularly (Lemaitre and Miguel-Aliaga, 2013), while in TSSMs, the remnants of plant cellular contents have been observed in vesicles of cellular origin, suggesting “intracellular” digestion (Wiesmann, 1968; Orlob and Takahashi, 1971; Mothes and Seitz, 1981). Furthermore, it is not clear if TSSMs have a peritrophic membrane or a hemolymph (Mothes and Seitz, 1981), further questioning mechanisms that underlie the distribution of acquired nutrients to different tissues.

The analysis of the TSSM genome revealed that its herbivorous pest adaptations at least partially associate with the expansion of gene families that encode proteins involved in digestion and detoxification (Grbić et al., 2011). Subsequent studies established that changes in the expression of these genes associate with TSSM host shifts (Dermauw et al., 2013; Zhurov et al., 2014; Wybouw et al., 2015), leading to the proposition that they are one of the main determinants of TSSM's xenobiotic responsiveness. However, a direct connection between gene family expansions and TSSM host adaptation has so far been demonstrated only in the case of cysteine synthase (Tu-CAS), which confers resistance of spider mites to cyanogenic glycosides (Wybouw et al., 2014). The establishment of forward (Van Leeuwen et al., 2012) and reverse (Khila and Grbić, 2007; Kwon et al., 2013, 2016; Suzuki et al., 2017b) genetic approaches should facilitate the understanding of the molecular basis of the TSSM digestive physiology, and mechanisms underlying host-adaptation and xenobiotic responsiveness. Although there have been several detailed ultrastructural studies that described the TSSM digestive tract (Mothes and Seitz, 1981; Andre and Remacle, 1984; Mothes-Wagner, 1985), a more comprehensive understanding of the TSSM digestive system is needed to support the functional analysis of its digestive and detoxification physiology. Here, we describe the organization of the TSSM digestive tract, complementing previously published ultrahistological studies by putting cellular details in a broader tissue-organization context. In addition, we follow a variety of tracer dyes to define the properties of compartments within the alimentary system. This study provides a histological framework for the functional analysis of TSSM digestive and detoxification physiology, hallmarks of TSSM extreme polyphagy and xenobiotic responsiveness.

## Materials and methods

### Mite rearing

The London mite population was maintained on the bean, *Phaseolus vulgaris*, cultivar California Red Kidney (Stokes, Thorold, ON) for more than 10 years. Bean plants were grown in a peat–vermiculite growing mix (Pro-Mix® BX Mycorrhizae^TM^; Premier Tech, Rivière-du-Loup, QC). The mites and their host plants were maintained at 26°C, under 100–150 μmol m^−2^ s^−1^ cool-white fluorescent light and a 16/8 h (light/dark) photoperiod.

### Paraffin embedding and sectioning of mite tissues

Spider mite adult females were collected and fixed overnight at 4°C in 4% formaldehyde in 10 mM phosphate buffer saline (PBS), pH 7.4 with 1% (v/v) Triton X-100. Mites were washed twice in 10 mM PBS and were subsequently dehydrated in an ethanol series (10%, 30%; 50% and 70%; v/v in H_2_O). Further dehydration and paraffin embedding were performed in tissue processor (Leica ASP300TP). Embedded mites were sectioned on a microtome (Leica RM2255 Microtome) at a thickness of 5 μm. Sections were dewaxed in two 10 min changes of 100% xylene and were progressively rehydrated. The following histological dyes were used as general staining: 0.1% Safranin O (C.I. 50240; MilliporeSigma) and 0.05% Fast Green (FCF, C.I. 42053, MilliporeSigma), 1% hematoxylin (C.I. 75290, MilliporeSigma) solution and 1% eosin (C.I. 45400, MilliporeSigma), and Movats' pentachrome solution. Images were obtained using a Zeiss AxioCam Color HRc CCD Camera 412-312 and Zeiss Axioplan II microscope. Finally, 300 nM of DAPI (D1306, Invitrogen) mixed in 10 mM PBS was used to visualize nuclei under epifluorescence Zeiss Axioplan II microscope.

### Cryo-sectioning of mite tissues

Adult female spider mites were fixed as described above, then frozen in OCT compound (Tissue-Tek) and stored at −80°C. Sections of approximately 10 μm in thickness were cut using the Leica CM3050 S cryostat (Leica, Austria). Sections were stained with 0.5% Oil Red O (102419, MilliporeSigma).

### Semi-thin sectioning of mite tissues

Adult female mites were fixed in solution of 2.5% (v/v) glutaraldehyde in 100 mM sodium phosphate buffer at pH 7. After 24 h, mites were washed in 100 mM sodium phosphate buffer at pH 7 and dehydrated in a graded alcohol series: 25, 50, 75, 95, 100 and 100% (v/v in H_2_O), for 15 min in each solution. Mites were embedded in LR White resin (Electron Microscopy Science, USA) and were cured overnight at 55°C. Specimens were cut with a Reichert Ultracut S ultramicrotome (Leica, Austria) into 1 μm serial sections, using a glass knife. Sections were stained with toluidine blue, 0.5% (w/v) in 0.1% (w/v) Na_2_CO_3_, for 5 min on a slide warmer at 60°C and dried overnight at room temperature.

### DAPI staining of digestive cells, feces and guanine pellets

Digestive cells were dissected from adult female mites by disrupting one of the caeca using a fine tungsten needle. All gut content, including digestive cells, was released into a solution of 75% (v/v) glycerol with 10 μM of DAPI (D1306, Invitrogen) in 10 mM PBS. Images were captured using an epifluorescence Zeiss Axioplan II microscope fitted with a DAPI filter cube (350–400 nm excitation, 417–477 nm emission) and an AxioCam Color HRc CCD Camera 412-312. For the collection of feces and guanine pellets, a small square arena of about 1.5 cm^2^ was created on a microscope glass slide using a wet Kimwipe. 100 adult female mites from the general rearing population were collected mid-day and were allowed to excrete for 1 h. A solution of 75% glycerol and 10 μM of DAPI (D1306, Invitrogen) in 10 mM PBS, was applied to deposits. Slides were examined under an epifluorescence Zeiss Axioplan II microscope fitted with a DAPI filter cube. Images were taken using AxioCam Color HRc CCD Camera 412-312. Images captured were further refined using the mask layer option from Adobe Photoshop® to highlight nucleus in digestive cells (Figures 5C–H; overlay) and “screen” as a blending mode for the nucleus in feces (Figure 5K).

### Guanine, chlorophyll and digestive products autofluorescence

Guanine particles localized in digestive cells and the posterior midgut were visualized with confocal microscopy using an argon laser at a 543 nm wavelength excitation and a LP of 650 nm. Chlorophyll and its degradation products were visualized with a He-Ne laser using a 633 nm excitation wavelength and a LP of 650 nm. A red false color was used to show the chlorophyll and its degradation product while a green color was used to highlight guanine crystals. Epifluorescence microscopy was also used to visualize expelled guanine crystals, using UV light with a FITC filter cube (450–490 nm excitation, 515–586 nm emission). Images were taken with a Zeiss Axioplan II microscope using AxioCam Color HRc CCD Camera 412-312.

### Microsphere size exclusion assay

To assess the size range of particles that can be ingested by adult female spider mites, a device for delivering water (control) or an aqueous suspension of polystyrene fluorescent microspheres (FMs; 50 nm to 1 μm in diameter; Polysciences) was designed using a 0.2-mL tube cap filled with either 50 μL of water or 2.5% (w/v) aqueous suspension of FMs and covered with stretched Parafilm® (see Supplementary Figure S1). Ten newly molted adult female mites were placed per device and incubated for 24 h at 25°C. Fluorescent images were taken with a digital camera (EOS Kiss X7, Canon) installed on a Leica M205FA microscope fitted with a GFP filter (395-455 nm excitation, >480 nm emission) with an exposure time of 5 s (ISO: 200). Bright-field images were taken using the same system without filters with an exposure time of 1 s (ISO: 200).

### Size exclusion properties of ventriculus-posterior midgut connection

To test the filtration properties of the ventriculus-posterior midgut connection, polystyrene FMs of 50 nm (Polysciences) and a fluorescent tracer dye (Alexa Fluor 555, Thermo Fisher Scientific) were delivered using the method described above. In addition, fluorescein-labeled dextran of various sizes (500, 40, and 4 kDa, MilliporeSigma) were delivered to adult female mites as mixtures with 6% blue dye (erioglaucine; McCormick, Sparks Glencoe, MD) using the leaf coating method (Suzuki et al., 2017a). To ensure the intactness of fluorescein-labeled dextran, the fluorescein-12-UTP (Roche) was delivered as a control. In both delivery systems, mites were allowed to feed for 24 h and fluorescence was visualized using an epifluorescence Zeiss Axioplan II microscope fitted with a FITC filter cube. Images were taken using an AxioCam Color HRc CCD Camera 412-312.

### Phalloidin staining

Approximately 100 adult spider mite females were incubated in 150 μM phalloidin solution (Alexa Fluor® 546 Phalloidin, ThermoFisher Scientific, USA) in 10 mM PBS, overnight at 4°C (Jiang et al., 2007). Phalloidin-stained actin filaments were visualized using a Zeiss Axioplan confocal microscope (Carl Zeiss AG, Germany) with the following settings: 543 nm excitation, Filter: Ch1, LP 560 nm.

### Lumen movement within TSSM gut

To visualize the lumen movement in the TSSM gut, a solution of 50% (v/v) glycerol diluted in 10 mM PBS and 0.1% (v/v) Tween 20 was gently applied to the back of an adult female mite with a fine brush to improve the transparency of the cuticle. Movement within the TSSM gut was recorded using a Canon EOS Rebel T5i camera (Canon, Japan).

### Estimation of gene expression level enrichment in proterosoma

For estimation of gene expression level enrichment in proterosoma, we have retrieved previously published transcriptome data from the Sequence Read Archive (SRA SRP074404, BioProject PRJNA320686) (Jonckheere et al., 2016). Reads were mapped to the reference *T. urticae* genome using STAR aligner version 2.5.3a (Dobin et al., 2013), allowing only unique mapping, up to five mismatches per read mapped, a minimum intron size of 20 bp, and a maximum intron size of 15,000 bp, in per-sample 2-pass mode. Read counts were generated using HTSeq at the level of gene locus in “union” mode (Anders et al., 2015). Estimation of gene expression fold changes and associated adjusted (BH) *p*-values was conducted using edgeR (Benjamini and Hochberg, 1995; Robinson et al., 2010) for genes that were expressed at the level of at least 1 CPM in at least one sample library. This procedure resulted in 12,814 genes retained for subsequent analysis. Lists of genes implicated in digestion and detoxification were obtained from previous publications (Santamaría et al., 2015a; Jonckheere et al., 2016), and high quality manual annotation of spider mite genome (Grbić et al., 2011). Identification of MFS and lipocalin genes was based on the significantly assigned PFAM domains PF07690, and PF00061, PF08212, respectively (Finn et al., 2014).

## Results

### Terminology

TSSMs, like insects, have a complete digestive system that consists of a foregut, midgut and hindgut. We have adapted the terminology used by Mothes-Wagner (1985) to describe the TSSM digestive tract, as it takes into account the embryonic origin of gut tissues. Accordingly, the foregut and hindgut contain compartments that are derived from the embryonic ectoderm and are consequently lined with the cuticle, while the midgut that is derived from the embryonic endoderm does not have a cuticular lining. The foregut consists of the stylet, buccal cavity, pharynx, esophagus and esophageal valve, the midgut includes the ventriculus, caeca and posterior midgut, and the hindgut is comprised of rectum and anal slit (Figure 1D). For the histology of the TSSM digestive system, we analyzed transverse, longitudinal, and sagittal sections of adult female TSSMs. We prepared serial sections, with examples shown in Supplementary Figures S2–S4, that allowed for a reconstruction of the TSSM alimentary tract. As our analysis of the TSSM digestive system complements previous ultrahistological studies (Mothes and Seitz, 1981; Andre and Remacle, 1984; Alberti and Crooker, 1985; Mothes-Wagner, 1985), we integrated observations from previous studies in describing different parts of the TSSM alimentary tract.

### Foregut

#### Determination of the particle sizes ingestible by the adult female TSSM

**Figure 2:**
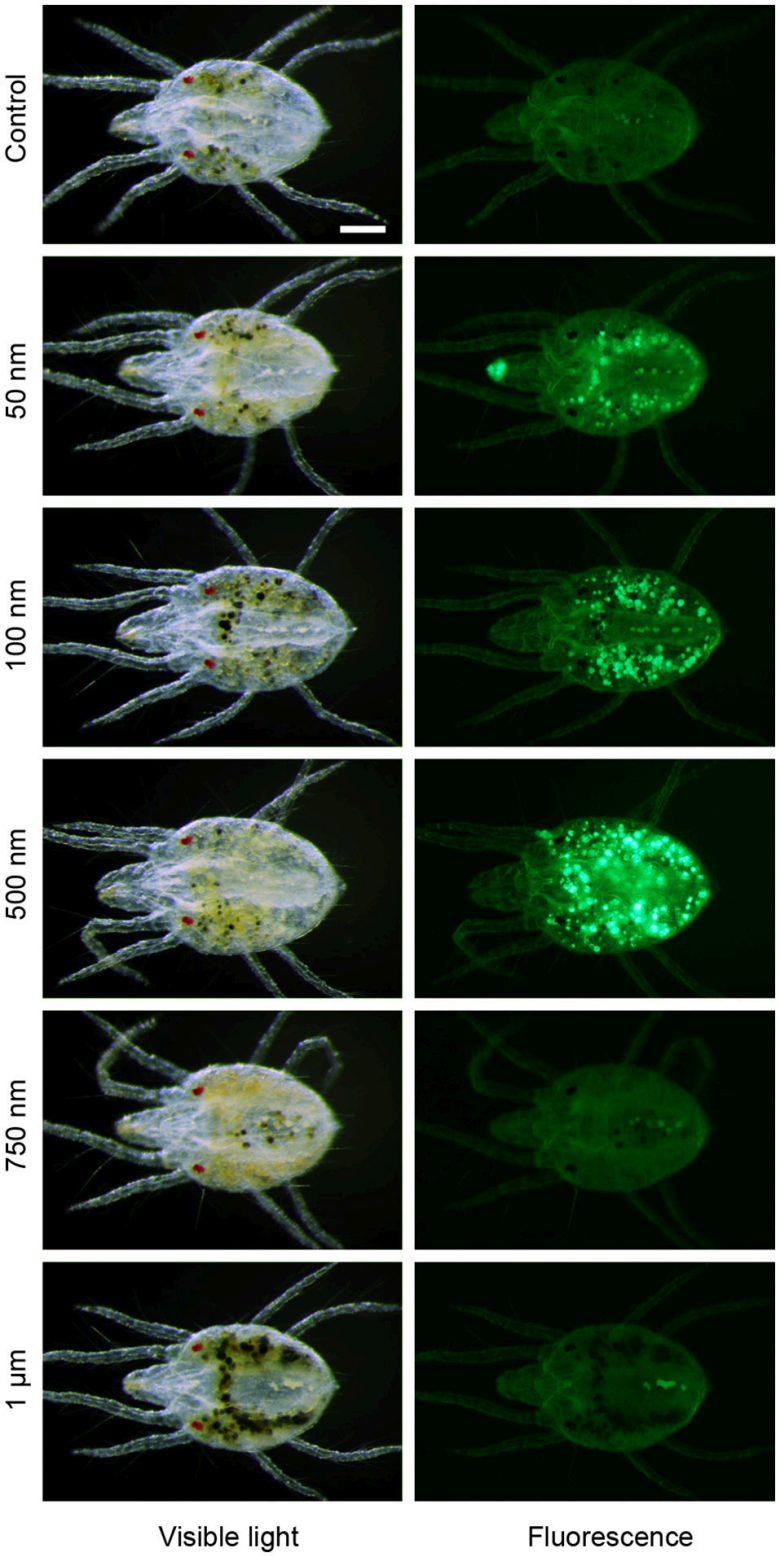
Size limitation of spider mite feeding apparatus. Accumulation of fluorescent microspheres of 50 nm to 1 μm in diameter in midgut of the spider mites after 24 h feeding at 25°C. Scale bar: 100 μm.

The first step in digestion of plant nutritive fluid within the TSSM alimentary tract is ingestion. Two scenarios for TSSM food ingestion were proposed: (i) TSSMs ingest food via the stylet (Summers et al., 1973; Hislop and Jeppson, 1976; Andre and Remacle, 1984; Bensoussan et al., 2016), (albeit connection of stylet and buccal cavity has not been demonstrated), or (ii) plant liquefied material is brought to the plant surface by capillary action where TSSM “laps” it into its buccal cavity (Alberti and Crooker, 1985; Nuzzaci and de Lillo, 1989, 1991). Regardless of the mode of nutrient uptake, the size of the ingestible particles is expected to be limited by the diameter of the stylet and/or the preoral groove/food canal. To determine the size cut off of ingestible particles, we used an aqueous suspension of polystyrene fluorescent microspheres (FMs) ranging in diameter from 50 nm to 1 μm. We designed a feeding device, shown in Supplementary Figure S1, where the liquid with FMs is sealed off by Parafilm®, requiring a TSSM to penetrate through the membrane with its stylet and to ingest the suspension. The mites' ability to ingest the FMs was assessed by detection of fluorescence within the digestive tract. As shown in Figure 2, FMs up to 500 nm in diameter accumulate in the midgut, however, no accumulation of fluorescent microspheres of 750 nm and greater was observed, indicating that TSSM can uptake only particles that are smaller than 750 nm. This is consistent with the size limitation imposed by the stylet and the preoral groove/food canal (Mothes and Seitz, 1981; Andre and Remacle, 1984; Nuzzaci and de Lillo, 1989, 1991).

#### Pharynx, esophagus

**Figure 3:**
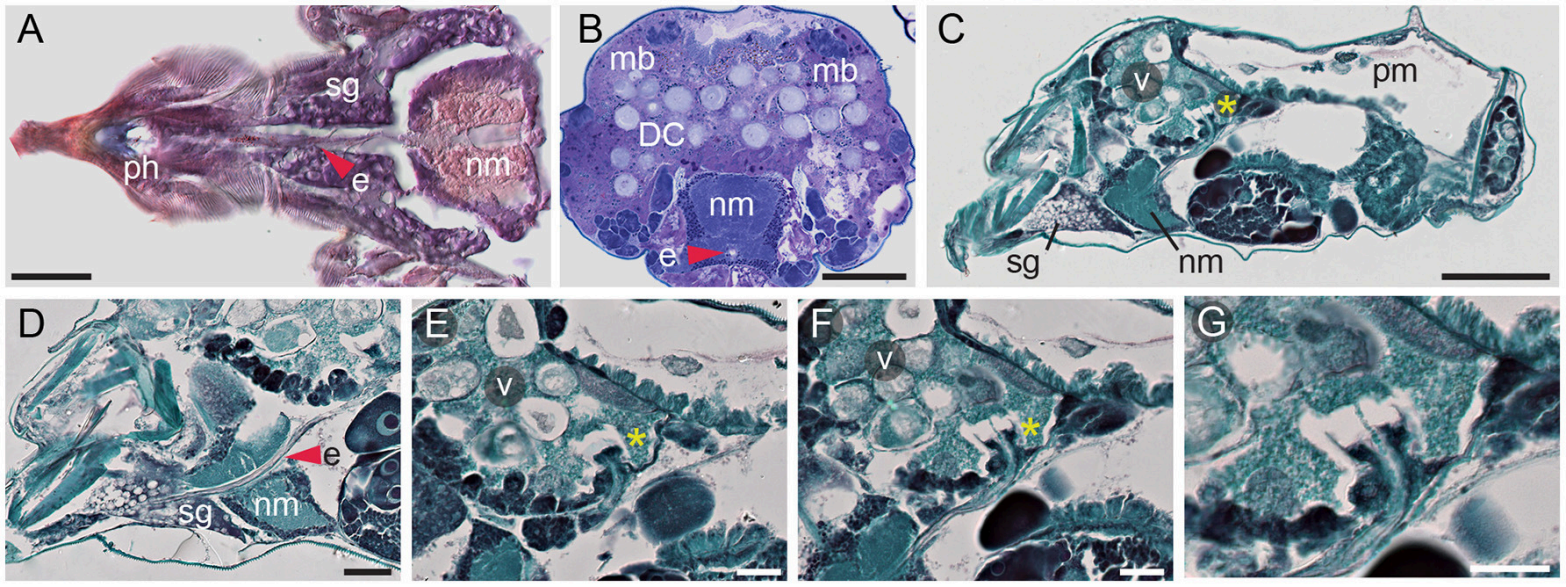
Esophagus and esophageal valve of spider mite. **(A)** Longitudinal cryosection of ventral part of proterosoma showing pharynx (ph) connected to esophagus (e) that passes through the silk gland (sg) and the nervous mass (nm) stained with Oil Red O. **(B)** Transverse section showing esophagus passing through nervous mass. Dorsal muscle bundles (mb) that facilitate midgut movement are clearly visible. LR White plastic embedding and Toluidine Blue staining. DC, digestive cells. **(C)** Sagittal section of female spider mite. Esophageal valve (asterisk) entering ventriculus (v). pm, posterior midgut. **(D)** Sagittal section through proterosoma of spider mite showing esophagus (red arrowhead) passing through silk gland and nervous mass toward ventriculus. **(E,F)** Consecutive sections through the esophageal valve (asterisk) entering the ventriculus. **(G)** Detail of esophageal valve entering ventriculus. Paraffin embedding and Fast Green and Safranin O staining were used in **(C–G)**. Scale bars: **(A)**: 25 μm; **(B)**: 50 μm; **(C)**: 100 μm; **(D)**: 25 μm; **(E–G)**: 20 μm.

On its way to the midgut, the ingested plant cell content passes through the pharynx and the esophagus (Figure 3). In longitudinal cross sections, the pharynx (ph) cavity is seen as an opening with dark-stained walls (Figure 3A, and Figure 3 in Mothes and Seitz, 1981), while the esophagus (e) is a tube that runs at median along the TSSM anterior-posterior axis (Figure 3). The esophagus initially passes ventrally through the silk glands (sg in Figure 3A) and then arches upwards as it traverses through the nervous mass (nm in Figures 3A–D). The esophagus enters into the ventriculus (v) of the midgut through the ventral layer of gut epithelial cells (Figures 3C,E–G) and terminates with the esophageal valve (asterisk in Figures 3C,E,F). The esophageal valve is cup-shaped and secures the inflow of the ingested plant content into the ventriculus of the midgut (Figure 3G). Ultra-high magnification cross sections through the esophagus, with clearly visible inner chitin lining, and the cup-shaped esophageal valve are also shown in Figures 11 and 12 in Mothes and Seitz (1981).

### Midgut

**Figure 4:**
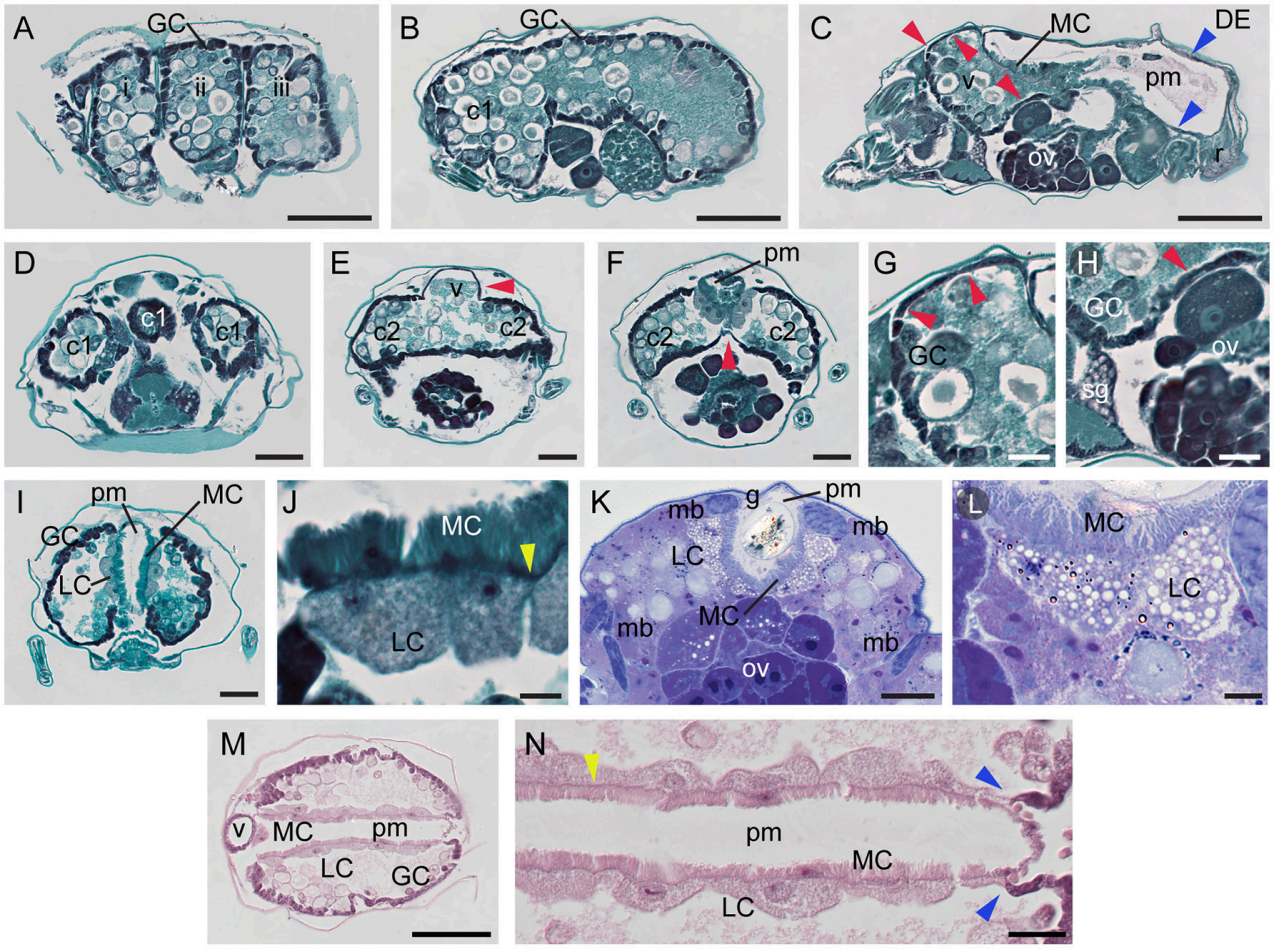
Midgut of spider mite. **(A–C)** Serial sagittal sections of spider mite female progressing from outside part of the body **(A)** showing lateral caecal pouches (i–iii) to the middle **(C)** where posterior midgut (pm) becomes visible. Red arrowheads point to the ventricular epithelium at cranial caeca and ventriculus (v). Blue arrowheads point to the thin single-layered squamous cells that lack microvilli projections at the dorsal and posterior region of the posterior midgut, DE. r, rectum. **(D–F)** Transverse sections through region of cranial caeca (c1), ventriculus (v), and anterior portion of posterior midgut (pm). **(G)** Detail of thin dorsal ventricular epithelium (red arrowheads) which transitions to cuboid generative cell (GC) epithelium anteriorly. **(H)** Detail of thin ventral ventricular epithelium (red arrowhead) adjacent to silk gland (sg) and ovary (ov). **(I)** Representative transverse section of mite digestive system showing generative cells (GC) of midgut epithelium, large cells (LC) of midgut epithelium adjacent to posterior midgut (pm), and microvilli cells (MC) of posterior midgut. **(J)** Large cells (LC) of midgut epithelium opposite of microvilli cells (MC) of posterior midgut epithelium. Yellow arrowhead points to basement membrane. **(K)** Transverse section of LR White plastic embedded and Toluidine Blue stained spider mite. **(L)** Large cells (LC) of midgut epithelium opposite of microvilli cells (MC) of posterior midgut epithelium. **(M)** Longitudinal section through ventriculus (v), midgut and posterior midgut (pm) stained with hematoxylin and eosin. **(N)** Detail of the interface between LCs of midgut and MCs of posterior midgut. Basement membrane (yellow arrowhead) is very well defined in this preparation. Notice the immediate change of cell types in both midgut and posterior midgut epithelia as two become separated in the posterior region (blue arrowheads). Scale bars: **(A–C)**: 100 μm; **(D–F)**: 50 μm; **(G,H)**: 20 μm; **(I)**: 50 μm; **(J)**: 10 μm; **(K)**: 30 μm; **(L)**: 10 μm; **(M)**: 100 μm; **(N)**: 20 μm.

The midgut (caeca, ventriculus, and the posterior midgut) comprises the greatest volume of the alimentary tract, filling almost the entire opisthosoma. Of five caeca, three project cranially (c1 in Figures 1B–D) and two are caudal, running laterally along the anterior-posterior axis of the TSSM body (labeled c2 in Figures 1B–D). Each caudal caeca forms three lobes (i, ii, iii), visible in Figures 1B–D, 4A. The caeca are interconnected through the ventriculus (v, Figures 1B–D, 4C). The ventriculus protrudes dorsally, above the caecal plane to form a separate compartment, Figures 1C, 4E. In addition, the ventriculus connects to the posterior midgut (pm) (Figures 1B–D, 4C), and thus, is a central hub that joins different parts of the midgut. The posterior midgut is the terminal part of the midgut that is nested dorsally between two caudal caeca (Figures 1B–D, 4 C,I,K,M). It enlarges and curves downwards at the posterior end (Figure 4C), where it transitions into the hindgut (rectum, r in Figures 1D, 4C).

**Figure 5:**
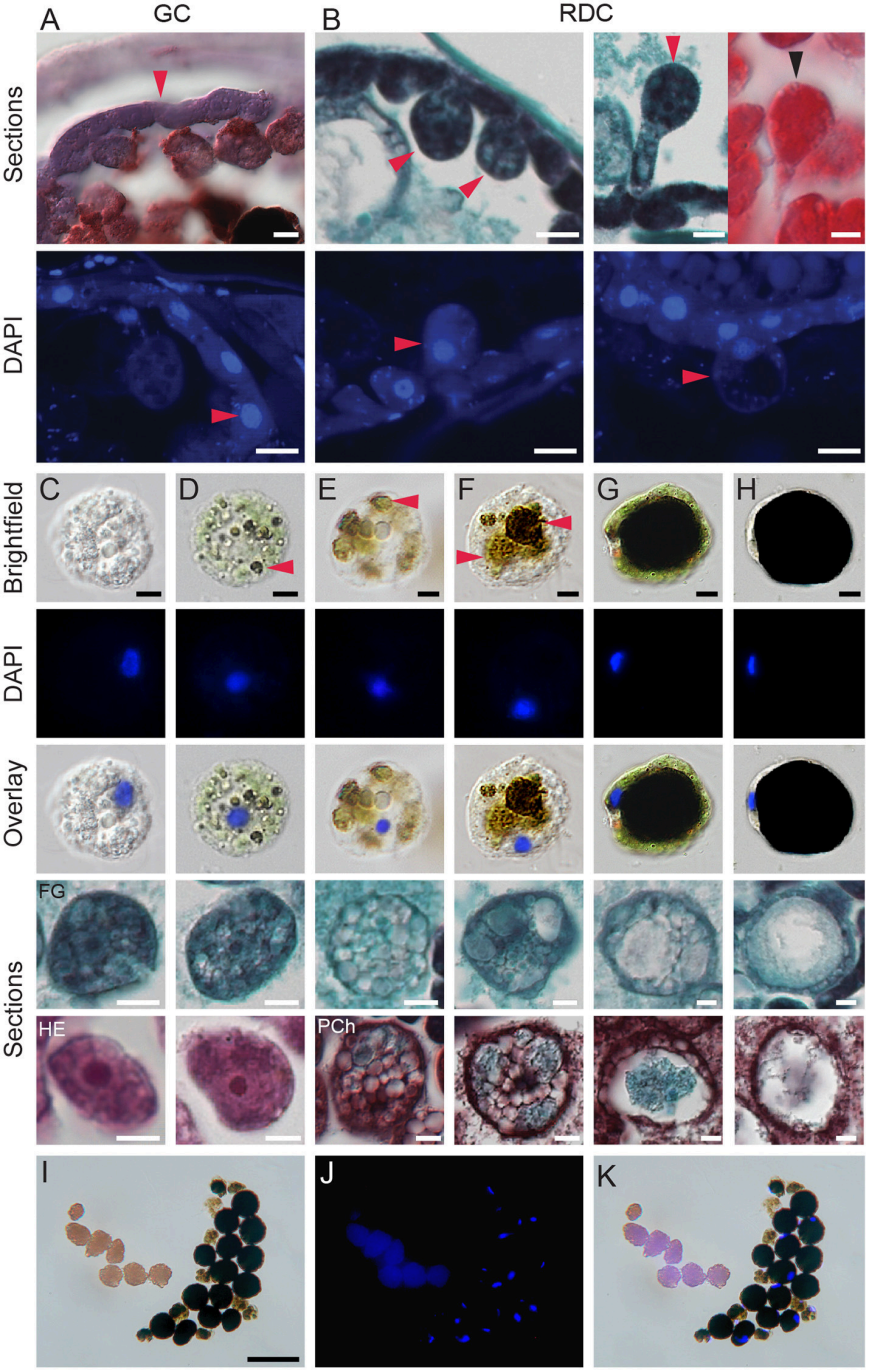
Development of digestive cells in spider mite midgut. **(A)** Brightfield microscopy (cryosection, Oil Red O staining) and fluorescence microscopy (paraffin sections, DAPI staining) of generative cells (GC) forming midgut epithelium. **(B)** Brightfield microscopy (paraffin sections, Fast Green and Safranin O, and Hematoxylin and Eosin staining) and fluorescence microscopy (paraffin sections, DAPI staining) of residual digestive cells (RDC) as they grow into the midgut lumen and detach from the midgut epithelial layer. **(C–H)** Digestive cells (DC) at various stages of development. DCs were dissected from the midgut lumen of female spider mites, stained using DAPI, and observed using brightfield and fluorescence microscopy. Lower panels, paraffin sections of DCs from different staining preparation: FG, Fast Green/Safranin O; HE, Hematoxylin/Eosin and Pch, Pentachrome. **(C)** Stage 1, the early stage of DC development with clear but numerous vesicles and large nucleus. **(D)** Stage 2, DC starting to uptake midgut lumen and plant pigments. Arrowhead marks colored vesicles within DC. **(E,F)** Stage 3, colored vesicles enlarge. **(G)** Stage 4, a large vesicle with dark content is surrounded by a thin layer of cytoplasm. Numerous small vesicles are still visible in cytoplasm, nucleus is displaced to the periphery. **(H)** Stage 5, the terminal stage of DC development characterized by a dense dark brown/black vesicle, a very thin crescent of cytoplasm and condensed nucleus on side of a DC. **(I–K)** Brightfield and fluorescence microscopy of spider mite feces. Feces are represented by guanine pellets and DCs at various stages of development. There is no apparent sorting of DCs based on developmental stage and amount of waste products accumulated. Guanine pellets readily absorb DAPI and also exhibit autofluorescence. Scale bars: **(A)**: 10 μm; **(B–H)**: 5 μm; **(I–K)**: 50 μm.

The midgut is comprised of a single-layered epithelium that encircles the midgut lumen. The midgut epithelium is composed of five cell types. In the caeca, three types of epithelial cells can be differentiated based on their shape. We refer to them as: (a) generative cells (GC, Figures 1D, 4A, B), (b) ventricular epithelial cells (VE in Figure 1D, red arrowheads in Figures 4C,E–H), and (c) large epithelial cells (LC) that form an inner wall of caudal caeca, adjacent to the posterior midgut (Figures 1D, 4I–N). GCs are the most abundant cell type in the midgut epithelium, building the outer midgut wall. They are cuboidal and densely stained in histological preparations. GCs detach from the epithelial wall and form free-floating vesicles in the midgut lumen (see below and Figure 5). No obvious histological differences were observed among the GC populations in different caeca. In addition, we did not observe any clear spatial patterning in the formation of the free-floating vesicles. Thus, it appears that GCs may all have generative capabilities. However, this should be tested in future experiments, e.g., by bromodeoxyuridine staining. Even though GCs form the majority of the caecal outer wall, there are few areas in the midgut outer epithelium that are composed of a different cell type—the ventricular epithelium (VE). The VE is located at the cranial caeca (Figures 4C,D,G), and in the dorsal (Figures 4E,G) and ventral (Figures 4F,H) areas of the ventriculus. VE cells form a thin squamous epithelium that does not detach into the lumen. The cranial caeca are in direct contact with head glands and head tissues (Mothes and Seitz, 1981), while the ventral VE is adjacent to developing ovaries/eggs (Figures 4F,H, Supplementary Figures S3, S4) and silk glands (Figure 4H). LCs are the third cell type that characterize the caecal epithelium. These are large, rounded cells that line the caudal caeca in contact with the posterior midgut (Figures 4I–N). In paraffin-embedded and sectioned tissue preparations, vesicles within the LCs are not readily visible. However, these vesicles are apparent in plastic-embedded specimens (Figures 4K, L, and Figure 22 in Mothes and Seitz, 1981).

The posterior midgut is seen as a V-shaped tube on transverse sections (Figures 4I,K). It runs along the dorsal midline, originating above the posterior portion of the ventriculus, and continues posteriorly above ovaries (Figure 4C) and between the caudal caeca (Figures 4M,N). At its anterior end, the posterior midgut connects to the ventriculus, while posteriorly, it enlarges and connects to the rectum (Figure 4C). The lateral walls of the posterior midgut are made of the fourth midgut cell type, squamous epithelial cells, with microvilli projecting into the lumen (MC in Figures 1D, 4C,I–N). These cells share the basement membrane (yellow arrowheads in Figures 4J,N) with the LCs that delimit the caeca. Posteriorly and dorsally, microvilli-containing cells are replaced with the flat cells that represent the fifth midgut cell type, DE (Figure 1D and blue arrowheads in Figures 4C,N). At the posterior transition of MC to DE cells, the caecal LCs are also replaced with GCs (Figures 4C,N), resulting in a coordinated change in the caeca-posterior midgut cellular superimposition.

The lumens of the caeca and the ventriculus are filled with large vesicles that were referred to as “food balls,” “floating cells” or “phagocytes” (Wiesmann, 1968; Mothes and Seitz, 1981; Alberti and Crooker, 1985). The lack of precision in the terminology describing these vesicles reflects the uncertainty of their origin and their fate within the TSSM digestive tract. Previous studies were not able to differentiate whether these vesicles arise from holocrine or apocrine secretions. In addition, while nuclei have been observed in some of these vesicles (for example see Figure 16 in Mothes and Seitz, 1981), they may not be present in others. Nevertheless, these vesicles have been firmly linked to the digestion of plant nutrients, as thylakoid and starch granules were observed within them (Orlob and Takahashi, 1971; Figures 16–20 in Mothes and Seitz, 1981). To determine the origin and fate of these vesicles within the TSSM digestive tract, we used a variety of approaches. In cryo-sectioned specimens, the GCs are strongly stained with pentachrome (Figure 5A, top). DAPI (4',6-diamidino-2-phenylindole) fluorescence staining of DNA (Figure 5A, bottom) confirms the presence of a single, large nucleus in each GC. GCs, while still attached to the basement membrane, enlarge and protrude into the caecal lumen (Figure 5B). They have nuclei and are referred to as Residual Digestive Cells (RDC). Ultimately, the RDCs detach from the epithelium and become free-floating cells, filling the lumen of caeca (Figures 1B,C, 3B, 4A–C). Free-floating cells dissected from the gut lumen were examined by brightfield microscopy (top row in Figures 5C–H), in DAPI-stained whole mount preparations (two middle rows in Figures 5C–H), and in paraffin sections (two bottom rows in Figures 5C–H). Our observations using the three visualization strategies suggest five stages in the development of the free-floating cells. In stage 1, upon detachment from the caecal epithelium, the cells are spherical and filled with many transparent vesicles (Figure 5C). In stage 2, most cells internalize the gut lumen content, resulting in the presence of two types of vesicles–transparent and pigmented ones (red arrowhead, Figure 5D). The internalization probably occurs through pinocytosis and/or phagocytosis as starch and thylakoid membranes had been observed within floating cells (Mothes and Seitz, 1981). In stage 3, the internal dark vesicles increase in size (Figures 5E,F). In stage 4, the cells have a single large vesicle with dense deposits that appear dark brown or black in bright-field optics. These deposits have been proposed to be waste products of digestion (Liesering, 1960; Wiesmann, 1968) (Figure 5G). The cytoplasmic ring is still visible in these cells and contains small vesicles. Lastly, in stage 5, the volume of the cell is almost completely occupied by the single dark-colored vesicle that is surrounded by a very thin cytoplasm (Figure 5H). Importantly, cells of all stages contain nuclei that are progressively flattened and displaced to the cell periphery as the central internal vesicle enlarges (see DAPI and Overlay rows in Figures 5C–H). Thus, we refer to these cells as digestive cells (DC). DCs of mixed stages are excreted as a fecal pellet. Even upon excretion, DCs stain with DAPI (Figures 5I–K), indicating that they retain nuclei and may be metabolically active.

### Functional anatomy of TSSM digestive tract

#### The filtration properties of the ventriculus-posterior midgut transition

**Figure 6:**
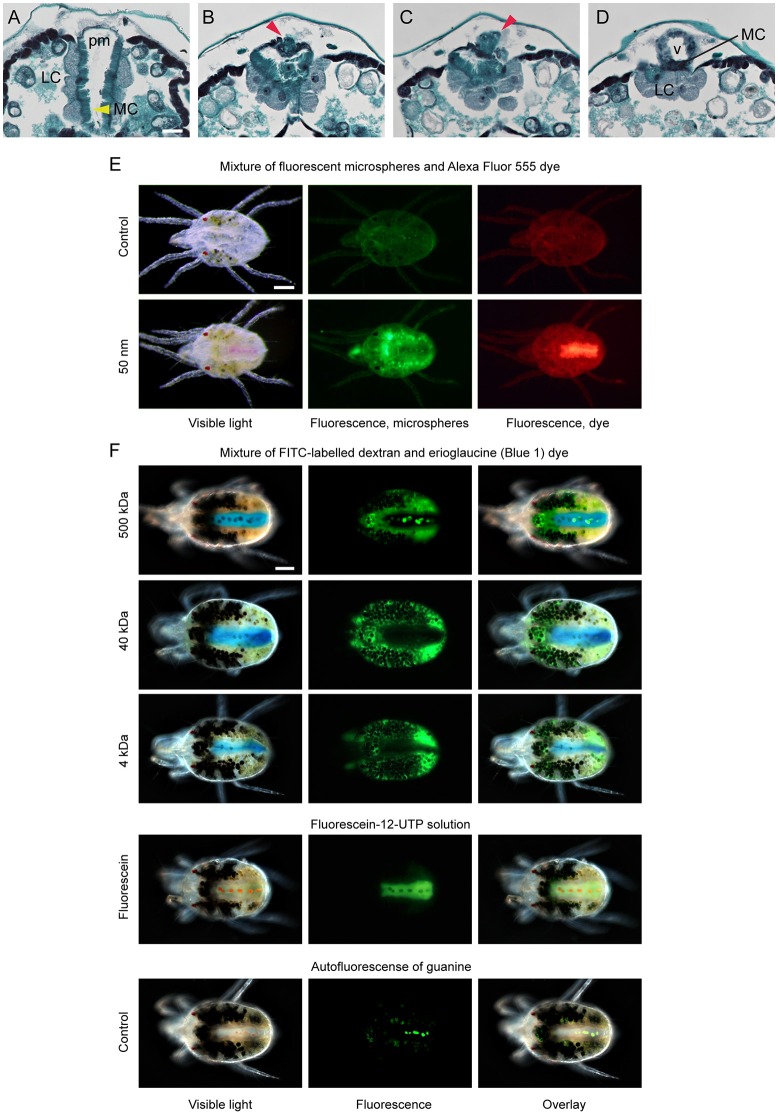
Permeability of ventriculus-posterior midgut barrier in spider mites. **(A–D)** Serial transverse sections of the dorsal region of spider mite body in the area of connection between ventriculus (v) and posterior midgut (pm). **(A)** Typical arrangement of V-shaped posterior midgut positioned between two caudal caeca. LC, large cells of caudal caeca epithelium adjacent to posterior midgut (pm); MC, microvilli cells of posterior midgut (yellow arrowhead). **(B,C)** Anteriorward sections through the posterior midgut that narrows, while the posterior-dorsal portion of the ventriculus appears (red arrowhead). **(D)** Section through the point of ventriculus-posterior midgut connection. **(E)** Distribution of the 50 nm fluorescent microspheres and fluorescent dye Alexa Fluor 555 (1250 Da) in mite midgut upon the ingestion of their mixture. Alexa Fluor 555 readily passes through midgut and accumulates within posterior midgut, while 50 nm microspheres are retained in the caeca lumen. **(F)** Distribution of the FITC-labeled dextrans of different sizes and erioglaucine dye in mite midgut upon the ingestion of their mixture. Erioglaucine (793 Da) and fluorescein-12-UTP (1034 Da) accumulate in posterior midgut while FITC-labeled dextrans (4, 40, and 500 kDa) are retained in the caeca lumen. Guanine autofluorescence can be seen within digestive cells in midgut and in pellets in the posterior midgut. Scale bars: **(A–D)**: 25 μm; **(E,F)**: 100 μm.

The caeca and the posterior midgut are two distinct compartments of the midgut that differ with regard to the continuous presence of digestive cells and epithelial cell types. Contraction of the paired dorsal longitudinal muscles positioned along the boundary between the caeca and the posterior midgut (marked as mb in Figures 3B, 4K, Supplementary Figure S5) creates caecal lumen movement (see Supplementary Movie). The ceacal lumen dynamically moves from one caudal caeca to another, readily seen by the movement of digestive cells. However, even though digestive cells pass through the ventriculus, they do not enter into the posterior midgut. Thus, the translocation of the material from the ventriculus into the posterior midgut seems to be regulated. A sphincter muscle (Figure 2 in Mothes-Wagner, 1985) was proposed to tightly control the passage from the ventriculus into the posterior midgut. We were unable to identify these muscles at the junction between the ventriculus and the posterior midgut in serial transverse sections or with confocal microscopy of phalloidin**-**stained tissues. However, the junction between the ventriculus and the posterior midgut can be seen in anteriorward serial sections shown in Figures 6A–D. In these sections, it is apparent that the posterior midgut terminates anteriorly in the complex that includes LCs from the caecal epithelium, the ventral edge of the posterior ventriculus, and microvilli cells of the posterior midgut epithelium (Figure 6D). Whether these epithelial cells and/or the sphincter muscle control the entrance to the posterior midgut is presently unknown. However, as the guanine and/or liquid excretion is frequent, it was proposed that water and small ions can transverse the ventriculus-posterior midgut barrier (McEnroe, 1961b). To test the permeability of this barrier we fed TSSMs with solutions containing a mixture of conjugated and free dyes, and followed their distribution within the TSSM digestive tract. Figures 6E,F show the distribution of the 50 nm fluorescent microspheres and Alexa Fluor 555 fluorescent tracer dye, and the 500 kDa fluorescein-conjugated dextran and erioglaucine (blue dye), respectively, when provided to TSSMs as mixtures. In both cases, the small molecules [Alexa Fluor 555 fluorescent tracer dye (Figure 6E) and erioglaucine (Figure 6F)] readily passed into the posterior midgut. On the contrary, the FMs and conjugated fluorescent dyes were retained in the caeca. This demonstrates the existence of differential filtering of the caecal lumen content by size, regulating entrance into the posterior midgut. To more precisely test the cut off size of the molecules that can enter into the posterior midgut, we tested the distribution of the 40 and 4 kDa fluorescein-conjugated dextran molecules that were each provided to TSSMs in a mixture with the erioglaucine. As seen in Figure 6F, both 40 and 4 kDa fluorescein-conjugated dextrans remained in the caeca, while erioglaucine passed into the lumen of the posterior midgut. Since fluorescein-12-UTP (Figure 6F), Alexa Fluor 555 fluorescent tracer dye and erioglaucine have molecular weights of 1.03, 1.25, and 0.8 kDa, respectively, these results indicate that the filtering cut-off between the ventriculus and the posterior midgut is between 4 and 1 kDa. It should be noted that the mobility of a particular compound does not only depend on its molecular weight, but also depends on its chemical properties. Regardless, these results indicate that small non-complex chemicals and metabolites can readily move from the ventriculus to the posterior midgut while more complex molecules, with higher molecular weights, such as proteins, will be retained in the caeca.

#### Excretion of nitrogenous waste

**Figure 7:**
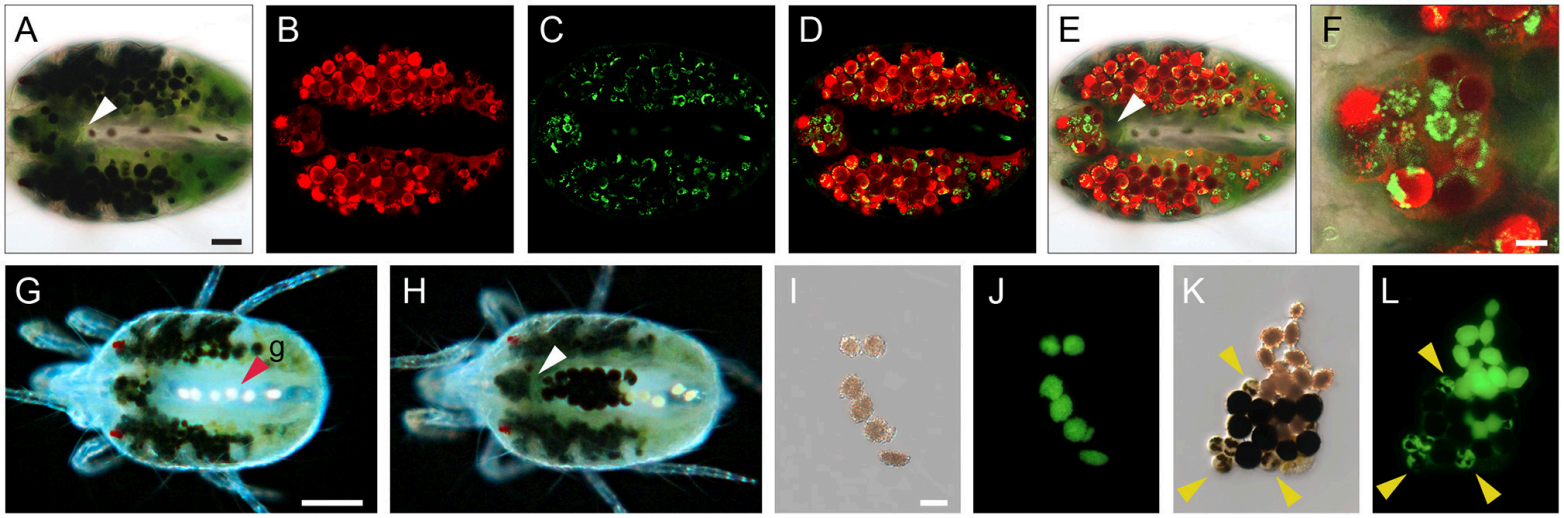
Compartmentalization of digestion and nitrogenous waste in spider mite. **(A)** Brightfield microscopy of a female spider mite showing characteristic spots produced by large numbers of mature DCs, green pigmentation of midgut lumen and colorless posterior midgut with a few guanine pellets within. White arrowheads point at the boundary between the midgut and the posterior midgut in panels **(A, E, H)**. **(B–D)** Confocal images of autofluorescence of **(B)** chlorophyll, red false color, **(C)** guanine, green false color, and **(D)** their overlay within mite midgut lumen, DCs and the posterior midgut. **(E)** Overlay of images in A–C. **(F)** Detail, showing distribution of chlorophyll (red) and guanine (green) in DCs in the ventriculus. **(G)** Guanine pellets (g, red arrowhead) within posterior midgut of female spider mite. **(H)** DCs and guanine pellets within posterior midgut of female spider mite. **(I)** Excreted guanine pellets. **(J)** Autofluorescence of guanine pellets under UV light. **(K)** Mixed excretion containing guanine pellets and DCs at various stages of development. **(L)** Autofluorescence of guanine pellets, and guanine deposits within DCs under UV light. Scale bars: **(A–E)**: 50 μm; **(F)**: 10 μm; **(G,H)**: 100 μm; **(I–L)**: 25 μm.

The Malpighian tubules mark the transition between the midgut and the hindgut in insect digestive tracts (Nation, 2015). They serve as an excretory organ that removes nitrogenous waste from the hemolymph and passes it to the hindgut for elimination, as part of the fecal excretions. TSSMs were reported to lack both the Malpighian tubules and the hemolymph (Blauvelt, 1945), raising the question of where is the nitrogenous waste formed and how is it disposed. TSSMs form guanine birefringent spherules that are disposed of as nitrogenous waste. They can be observed using brightfield (Figures 4K, 7A,G,H), confocal (Figures 7C–F) or epifluorescence (Figures 7J,L) microscopy. Guanine fluorescence is associated with two distinct forms that can be seen in the posterior midgut and caeca. In the posterior midgut, pellets of approximately 20–30 μm are made exclusively of guanine (g, Figures 1D, 4K and arrowhead in Figure 7G). The guanine pellets are excreted as guanine fecal droplets alone or together with digestive cells (Figures 7G–L). In the caeca, guanine particles are smaller (Figure 7C). The overlay of chlorophyll (associated with DC phagocytosis of the ingested plant cell content) and guanine fluorescence indicates that nitrogenous waste accumulates within digestive cells (Figures 7D,E and close up in Figure 7F showing individual guanine particles within DCs). The guanine within the digestive cell is excreted along with the chlorophyll waste products (for example see DCs labeled with arrowheads in Figures 7K,L). Note that neither LCs of the caecal epithelium nor microvilli-bearing cells (MC) forming the lateral walls of the posterior midgut show any fluorescence (Figure 7E), suggesting that they may not be involved in the digestion of plant cell contents and do not accumulate guanine waste products. It is currently unknown how guanine pellets form in the posterior midgut, yet they are the most abundant form of fecal deposits. Over the course of 1 h, we observed that a population of 100 mites excretes an average of 102 ± 13 guanine-only fecal pellets and an average of 12 ± 3 fecal pellets that contain both DCs and guanine (*n* = 4, mean ± SD reported).

### Expression pattern of the digestion/detoxification related genes

**Figure 8:**
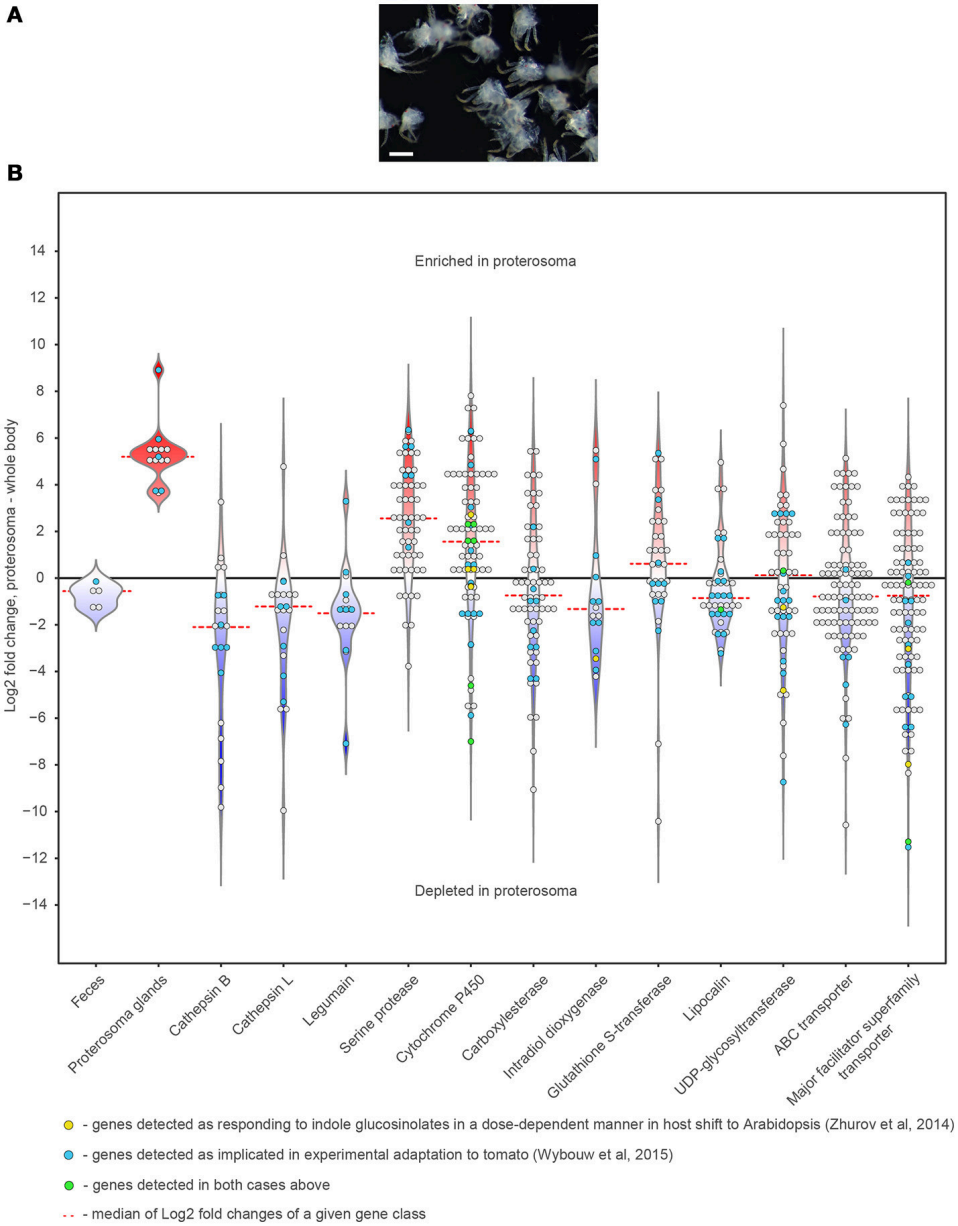
Expression patterns of the digestion/detoxification related genes within spider mite body. **(A)** Dissected spider mite proterosomas used for the RNA extraction. **(B)** Violin and dot plots showing distribution of Log2 Fold Changes of expression of genes implicated in digestion and detoxification in mite proterosoma compared to the whole-body levels of expression. Scale bar: 100 μm.

Anatomical characterization of the TSSM digestive system provides a cellular framework for the functional characterization of processes associated with the digestion of plant nutrients and detoxification of plant defense compounds. Gene families associated with digestion and detoxification underwent a significant expansion in the TSSM genome (Grbić et al., 2011) and were repeatedly associated with TSSM-plant interactions and pesticide detoxification (Dermauw et al., 2013; Zhurov et al., 2014; Wybouw et al., 2015). To identify genes within these families that have roles in the TSSM gut, we investigated their expression domains through comparisons of transcriptome data from mite proterosoma to whole-body data of bean-reared TSSMs (Figure 8). Expression of genes encoding proteins detected in the feces (and thus associated with TSSM gut) (Santamaría et al., 2015a) and salivary glands (Jonckheere et al., 2016), were used as controls to calibrate gene expression depletion and enrichment patterns in the proterosoma, respectively (Figure 8B). Peptidases associated with digestive processes in mites such as cathepsins-B and -L, and legumains, were generally strongly depleted in the proterosoma, consistent with their predicted localization in gut cells. Similarly, carboxylesterases, intradiol ring-cleavage dioxygenases, lipocalins, and transporters showed a pattern that is consistent with their greater expression in the TSSM gut. By contrast, the expression of serine proteases, which have not been implicated in mite digestive processes, was found to be enriched in the proterosoma, as were most cytochrome P450 genes. When genes associated with experimental mite adaptation to tomato (Wybouw et al., 2015) or with TSSM responsiveness to *Arabidopsis* indole glucosinolate defense compounds (Zhurov et al., 2014) were highlighted within the classes of analyzed genes, a majority (89 out of 135 genes) corresponded with genes depleted in the proterosoma and potentially associated with the digestive system. However, approximately 1/3 of xenobiotic-responsive genes mapped to the proterosoma in mites reared on beans. Whether these genes undergo tissue-specific changes in their expression, as suggested by this analysis, is at present not known but should be checked by *in situ* localization of their expression in xenobiotically-challenged mites.

## Discussion

Using a variety of histological techniques in combination with tracer dye experiments, we analyzed the organization of the TSSM digestive tract as well as the functional properties of digestive compartments relative to their ability to parcel out molecules of different molecular weights. This study lays a histological basis for a better understanding of TSSM-plant interactions at the cellular level. Here, we discuss features of TSSM digestive physiology that are expected to profoundly affect the interaction between the TSSM and its plant hosts.

### The role of stylet in TSSM digestive physiology and in modulation of plant-mite interactions

The stylet is composed of two cheliceral digits that interlock when protracted, forming a hollow tube-like organ (Andre and Remacle, 1984). Mites protrude the stylet during feeding (Beard et al., 2012; Bensoussan et al., 2016), but withdraw it upon any perturbation. As a consequence, histological studies addressing the involvement of the stylet in plant nutrient ingestion were performed on mites with withdrawn stylets, making the resolution of stylet connectivity with the buccal cavity/pharynx unclear (Summers et al., 1973; Jeppson et al., 1975; Andre and Remacle, 1984; Nuzzaci and de Lillo, 1991; see discussions in Alberti and Kitajima, 2014b; Bensoussan et al., 2016). We have previously captured mites in a feeding position, with their stylets inserted in the plant cells from which they are feeding (Bensoussan et al., 2016). The invariable association of stylets with feeding cells in our preparations is consistent with the possibility that mites use stylets to ingest plant cell content. Here we showed that there is a size limit to particles TSSMs can ingest through their feeding apparatus. Their exclusion size, smaller than 750 nm (Figure 2), is consistent with the inner stylet diameter of 1 μm (Andre and Remacle, 1984). Furthermore, the stylet was implicated in the delivery of salivary secretions: (1) stylet is directly connected to the salivary glands (de Lillo et al., 2002), and (2) salivary secretions were detected in a solution that was enclosed in a parafilm bubble accessible only by stylets (Jonckheere et al., 2016), in a similar experimental set up that was used in the feeding experiment shown in Figure 2. If TSSMs use stylets to ingest plant nutrients, then the stylet is expected to transport material both inwardly (the plant cell content) and outwardly (the salivary secretions). This is in contrast to other cell-content feeding arthropods that show more specialized stylet structures. For example, Eriophyoid mites have nine stylets that are individually used for piercing, salivary injections, and nutrient intake (Krantz and Lindquist, 1979). In addition, even though phloem-feeding arthropods such as aphids have a single stylet, their stylets consist of two canals–one that acts as a conduits for salivary secretions and one that allows the ingestion of phloem sap (Tjallingii and Esch, 1993; Garzo et al., 2012). How and whether these anatomical differences affect TSSMs' ability to deliver and potentially take up solutions is not clear. Thus, a further functional understanding of the TSSM feeding apparatus is required to identify TSSM-specific adaptations that support its feeding style.

The identification of the cut off size of ingestible particles is strongly suggestive of liquefaction and partial digestion of plant cellular content prior to its intake by TSSMs (Mothes and Seitz, 1981). Enzymes involved in the preoral digestion of plant cell content may originate from the vacuole of the pierced plant cell, which is expected to burst upon stylet penetration. In addition, digestive enzymes (e.g. cathepsins, serine proteases, glycoside hydrolases, beta-galactosidases, and beta-mannosidases) together with proteins of unknown functions were identified in TSSM salivary secretions (Jonckheere et al., 2016, 2018). Thus, TSSM feeding is associated with partial digestion and decomposition of plant structures, and the presence of TSSM-secreted proteins, generating a rich source of potential Damage and Herbivory Associated Molecular Patterns (DAMPs and HAMPs), some of which can act as elicitors of plant defenses. It has been shown that TSSM salivary secretions also contain effector proteins (Villarroel et al., 2016). Even though functional analysis of TSSM secreted proteins is at its infancy, it is expected that TSSMs have a wide repertoire of effectors that can interfere with plant responses. Thus, the stylet provides an interface between the TSSM and the host plant; as mites feed, they cause cellular damage and contribute elicitors and effectors at the feeding site. On the other hand, plants perceive DAMPs, HAMPs and other stimuli that trigger their responses to mite herbivory. The balance between TSSM- and plant-derived compounds ultimately determines the nature and the intensity of plant defenses.

### Digestion of plant nutrients

Digestion of plant nutrients in the TSSM gut is another process in which plant and TSSM compounds interact. However, as the TSSM digestive system departs from the canonical insect alimentary tract, it is not clear if, how and where these anti-digestive plant compounds act to reduce TSSMs' ability to retrieve nutrients.

Ingested liquefied plant cell content passes through the esophagus to reach the ventriculus within the TSSM midgut. The esophageal valve enters the ventriculus ventrally and releases the ingested food into the caecal lumen (Figure 3). We demonstrated the existence of a regulatory system between the ventriculus and the posterior midgut, capable of separating molecules based on size. Using mixtures of conjugated dextran or fluorescent microspheres, and small tracer dyes, we showed that molecules smaller than approximately 1.25 kDa pass to the posterior midgut, while molecules greater than 4 kDa are retained in the caecal lumen (Figure 6). Thus, the ingested mixture of plant cell content can be rapidly separated into a fraction that passes to the posterior midgut (corresponding by size to e.g. ions and plant primary and secondary metabolites), and complex biological molecules that are retained in the caecal lumen (including proteins and remnants of plant cellular structures) (Figure 6). Molecules retained in the caecal lumen undergo digestion.

It has been suggested that unlike in insects, digestion in some Acari occurs (at least partially) intracellularly (Sojka et al., 2013). In TSSMs, the midgut lumen is filled with digestive cells that originate from the midgut epithelium. Previously, their ontogeny has been attributed to apocrine or holocrine secretions of the midgut epithelium cells (Mothes and Seitz, 1981). However, we showed that these cells retain their integrity, including the nucleus, throughout their lifespan–from detaching from the midgut epithelium to their final excretion in feces (Figure 5). Furthermore, the cytoplasm of these cells is filled with small vesicles that gradually gain the color of the midgut lumen and enlarge, to reach a final state where a thin cytoplasmic layer encircles the large vesicle that appears dark brown/black in bright-field optics. Observation of starch granules and thylakoid membranes in these cells (Mothes and Seitz, 1981), and the identification of dark deposit as a breakdown product of plant pigments (Liesering, 1960; Wiesmann, 1968) strongly suggest that these cells take up the gut lumen content (i.e. ingested plant material) via pinocytosis or phagocytosis and digest it. We referred to them as digestive cells (to avoid confusion of previously used term “phagocytes” that implicates immune function), even though a direct demonstration that they actively carry the digestion process is still missing. Aspartyl and cathepsin L-like proteases have been identified in TSSM feces, implying that they are the most abundant digestive proteases in the TSSM gut (Santamaría et al., 2015a). If lumen-floating cells participate in digestion, then aspartyl and cathepsin L-like digestive proteases should localize in vesicles within these cells, a prediction that should be tested by immunolocalization. In the tick *Ixodes ricinus*, it has been shown that digestive proteases localize in numerous vesicles within digestive cells (Sojka et al., 2013), unequivocally demonstrating that ticks digest hemoglobin intracellularly. In addition, in the herbivorous false spider mite, *Brevipalpus phoenicis*, intracellular digestion is implied, as its midgut is filled with compacted cells and the lumen is reduced to a system of small clefts (Alberti and Kitajima, 2014a). In contrast, in Acari that feed on solid food, like *Acarus siro* and *Archegozetes longisetosus*, ingested food particles form a food bolus (peritrophic membrane/matrix enveloped food particles) whose digestion is at least initially extracellular and restricted to the gut lumen (Alberti et al., 2003; Šobotník et al., 2008).

In response to herbivory, plants synthesize anti-digestive enzymes that are best characterized in the tomato (Gu et al., 1999; Chen et al., 2004, 2007; Fowler et al., 2009; Chung and Felton, 2011; Gonzales-Vigil et al., 2011). These enzymes include protease inhibitors that interfere with the action of digestive proteases, and amino acid-degrading enzymes that eliminate digested nutrients. Plant-host triggered protease inhibition has been demonstrated in the TSSM (Santamaria et al., 2012; Santamaría et al., 2015b; Santamaria et al., 2018). However, even though the efficacy of tomato anti-digestive enzymes in restricting TSSM fitness has not been tested, the predicted pH range of TSSM digestive compartments of 3.5–5.5 (Santamaría et al., 2015a) may not be conducive for their activity.

### Distribution of nutrients and excretion of waste products

During digestion, plant macromolecules are broken down to release nutrients needed to support TSSM metabolism. Egg and silk production are the greatest sinks for released nutrients, as female mites lay 2–10 eggs per day (egg diameter being approximately 1/5 of female body length) (see Figures 1C,D) and continuously deposit silk while moving (Saitô, 1977). In insects, the distribution of nutrients occurs through the hemolymph circulating within the hemocoel that is in direct contact with the sink tissues (Nation, 2015). In addition, insects store nutrients in fat bodies in the form of glycogen and lipid droplets (Arrese and Soulages, 2010). However, TSSM tissues are compact (see Figure 3B, Supplemental Figure 5A for examples) and no cavity that could correspond to the hemocoel could be observed. Likewise, mites do not have fat bodies (Mothes-Wagner, 1982). This raises the question of how nutrients are distributed and stored in TSSMs. The ventricular epithelium (patches of thin cells, Figures 4E–H) is in direct contact with sink tissues, indicating that it may be involved in transport of nutrients. However, if digestion is occurring in digestive cells that float in the caecal lumen, then it is unclear how nutrients reach ventricular cells or any other epithelial cells. Alternatively, nutrients may be absorbed in the posterior midgut by the epithelial cells with microvilli, as suggested by Mothes-Wagner (1985), who observed both glycogen depositions and lipid droplets in these cells. If MCs acquire nutrients from the lumen and/or directly from digestive cells as they pass through the posterior midgut, then the transport of nutrients from these cells to sink tissues is likely indirect, as they are not in a direct contact.

Furthermore, remnants of undigested food and byproducts of digestive processes also accumulate in the gut. Guanine, the end product of nitrogen metabolism (McEnroe, 1961a; Wiesmann, 1968) is easily detected due to its refractive (Figure 4K) and fluorescent properties (Figure 7). Guanine accumulates in digestive cells and large guanine conglomerates form in the posterior midgut (Figures 5, 7, and Occhipinti and Maffei, 2013). Guanine crystals are the most frequent excretory particles, yet the location of their synthesis is still not known. It was proposed that the caecal epithelium adjacent to the posterior midgut (LCs) might synthesize guanine, as these cells have an abundance of vesicles with excretory depositions (Mothes and Seitz, 1981; Mothes-Wagner, 1985). However, these cells do not show guanine fluorescence under fluorescence or confocal microscopy (Figure 7E). Alternatively, it was proposed that guanine forms in the caecal lumen and accumulates in the ventriculus from which it is passed to the posterior midgut (Occhipinti and Maffei, 2013). However, we observed no guanine conglomerates in the midgut lumen outside of DCs. In fact, DCs that are excreted in fecal pellets still contained guanine (Figure 7). Finally, posterior midgut epithelial cells with microvilli may contribute to the formation of these crystals, as proposed by Mothes and Seitz (1981). However, these cells also lack fluorescence under fluorescence or confocal microscopy (Figure 7E). The resolution of the guanine biosynthetic pathway and *in situ* localization of transcripts/proteins of biosynthetic enzymes should help resolve the management of nitrogen metabolism in TSSM.

### Detoxification of xenobiotic compounds

Adaptation to xenobiotic compounds encompasses many strategies including avoidance, target site modification, metabolism/detoxification, transport/excretion and sequestration (Després et al., 2007). The existence of molecular size regulation in the form of a “shunt” between the ventriculus and the posterior midgut (Figure 6) predicts that ingested plant secondary metabolites, the majority of which have a molecular weight < 1 kDa, will go directly to the posterior midgut and be excreted. In contrast, insect alimentary canals are linear, forcing all ingested compounds to pass through the whole length of the gut. The ability of TSSMs to expedite small molecules for rapid excretion potentially reduces the accumulation of allelochemicals in the midgut and may contribute to TSSM robustness to xenobiotic exposure. However, some plant toxins, despite being of small size, have chemical properties (e.g. are lipophilic) that allow them to be retained in the TSSM gut (e.g. enter cells through passive diffusion). TSSM resistance to such allelochemicals is likely based on detoxification. The analysis of the TSSM genome identified major proliferations of gene families encoding enzymes involved in oxidation, hydrolysis and/or reduction, and conjugation (Grbić et al., 2011). In addition, studies of TSSM transcriptional changes induced by either a host-shift or the exposure to pesticides established that the expression of detoxification genes changes in response to a xenobiotic challenge (Dermauw et al., 2013; Zhurov et al., 2014; Wybouw et al., 2015). In insects with sufficiently large body sizes, transcriptional analysis of xenobiotic responsiveness was performed directly on dissected gut tissues. For example, host-specific transcriptional reprograming of midgut physiology has been described in *Trichoplusia ni* (Herde and Howe, 2014). In addition, the analysis of dissected gut compartments of *Anopheles gambiae* larvae revealed that even though the expression of detoxification genes occurs throughout the gut, detoxification functions dominate in the anterior midgut and the hindgut/Malpighian tubes (Neira Oviedo et al., 2008). This is consistent with the discrete patterns of expression of cytochrome P450 genes in Drosophila, where they localize in the fat body, Malpighian tubules, and in the midgut (Chung et al., 2009). TSSMs are lacking the fat body and Malpighian tubules. In addition, TSSMs are small and thus not readily amenable to dissections of individual tissues. As a result, tissues/cells that are responsive to xenobiotic challenge have not been determined and are still unknown.

Here, we described how the TSSM digestive physiology deviates from the canonical processes as characterized in insects. This work builds on earlier ultrastructural analyses of the TSSM gut and provides a cell biology context for TSSM-plant interaction studies. Combining this knowledge with genomic and reverse genetics tools will allow a functional dissection of the TSSM gut and the identification of the specific features that enabled the evolution of an extreme generalist feeding strategy characteristic of two-spotted spider mites.

## Author contributions

NB, VZ, TS, MG, and VG conceived and planned the study. NB, CO, and SY performed experimental procedures and collected data. NB, VZ, TS, MG, and VG performed analysis and wrote the manuscript.

### Conflict of interest statement

The authors declare that the research was conducted in the absence of any commercial or financial relationships that could be construed as a potential conflict of interest.
