# Supplementary figures and images for: The Digestive System of the Two-Spotted Spider Mite, Tetranychus urticae Koch, in the Context of the Mite-Plant Interaction
Source: Front Plant Sci. 2018 Sep 11;9:1206. doi: 10.3389/fpls.2018.01206 (PMC6142783; doi:10.3389/fpls.2018.01206)

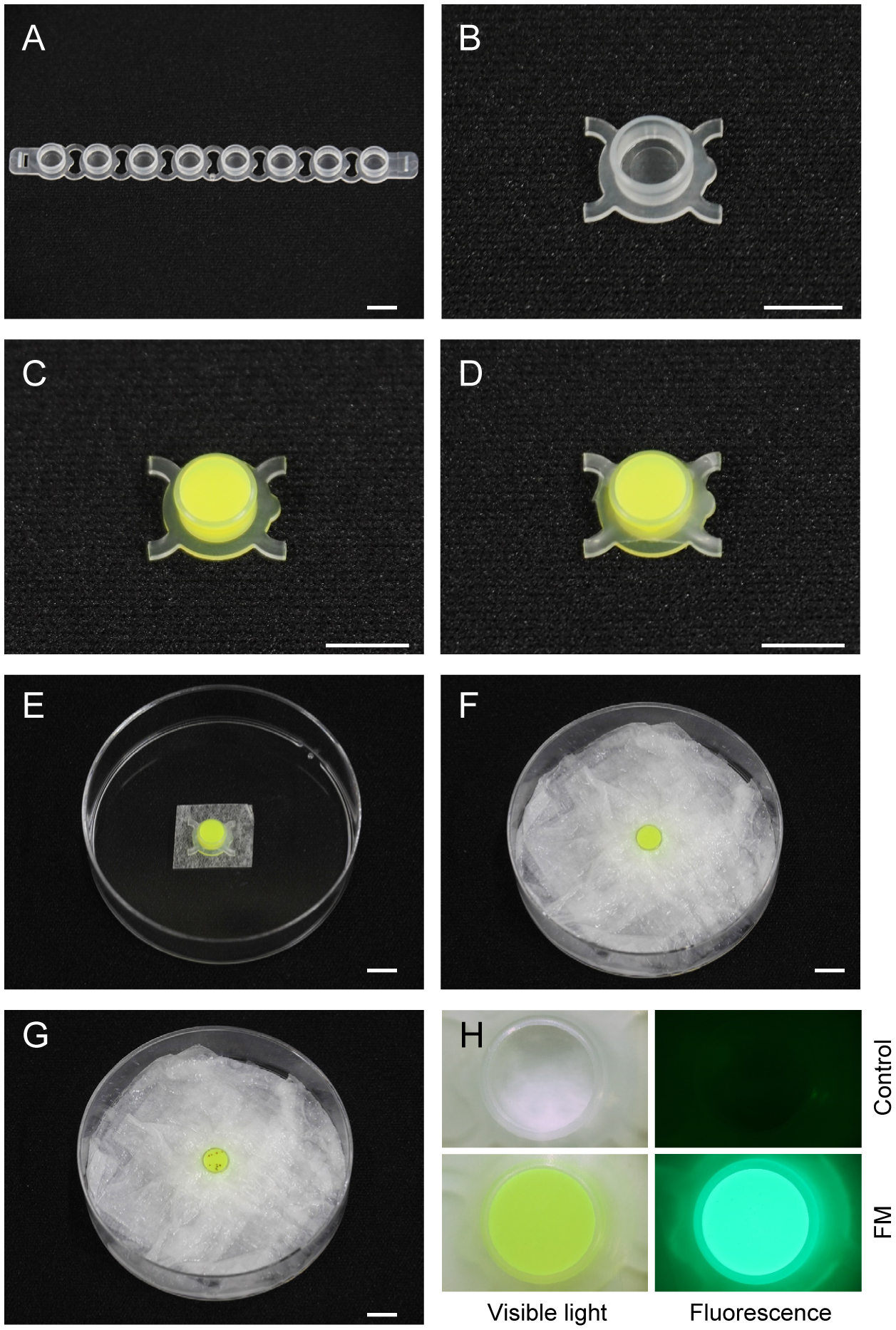

Supplement: Supplementary file 2 [file Image_1.tif]

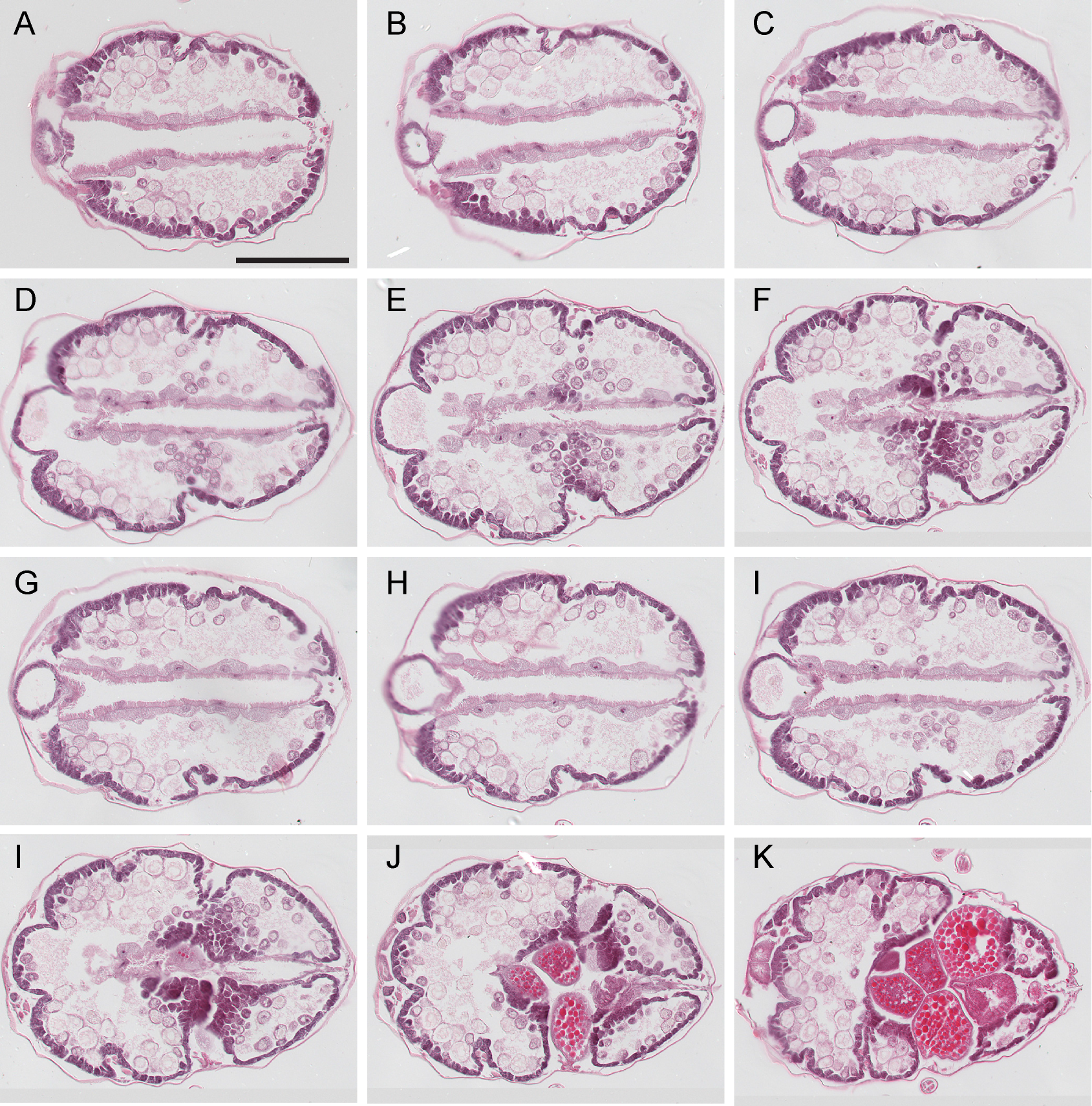

Supplement: Supplementary file 3 [file Image_2.TIF]

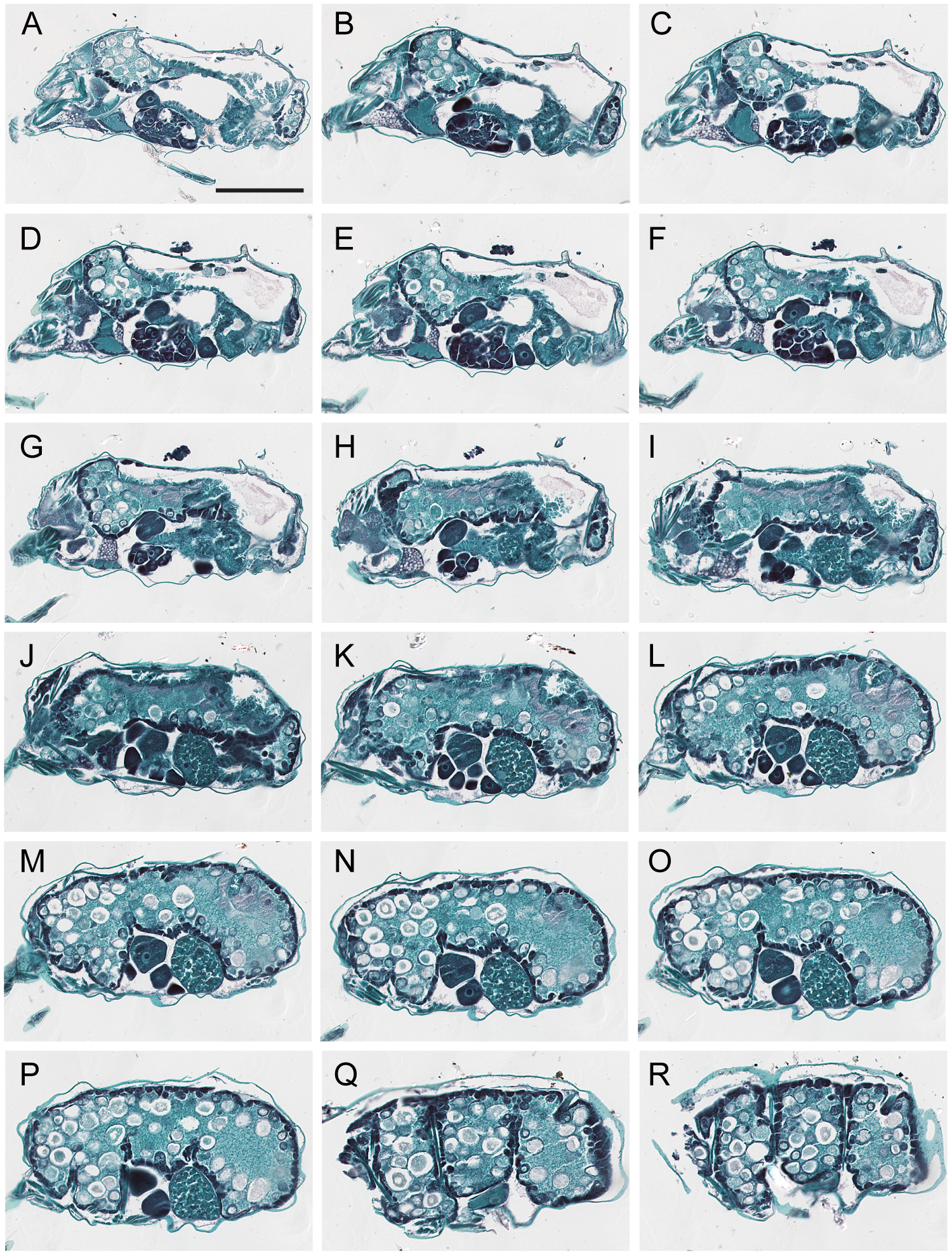

Supplement: Supplementary file 4 [file Image_3.TIF]

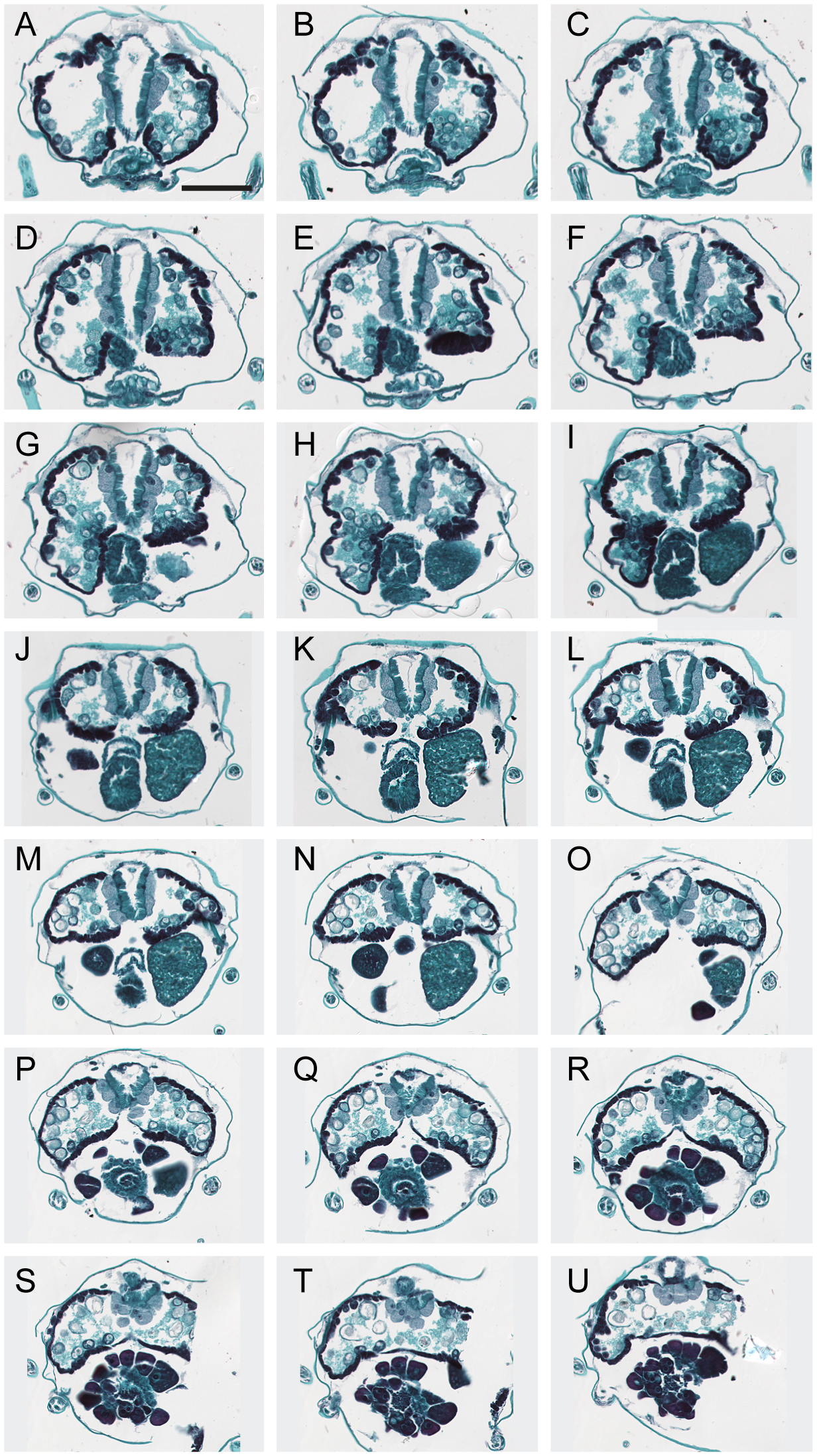

Supplement: Supplementary file 5 [file Image_4.TIF]

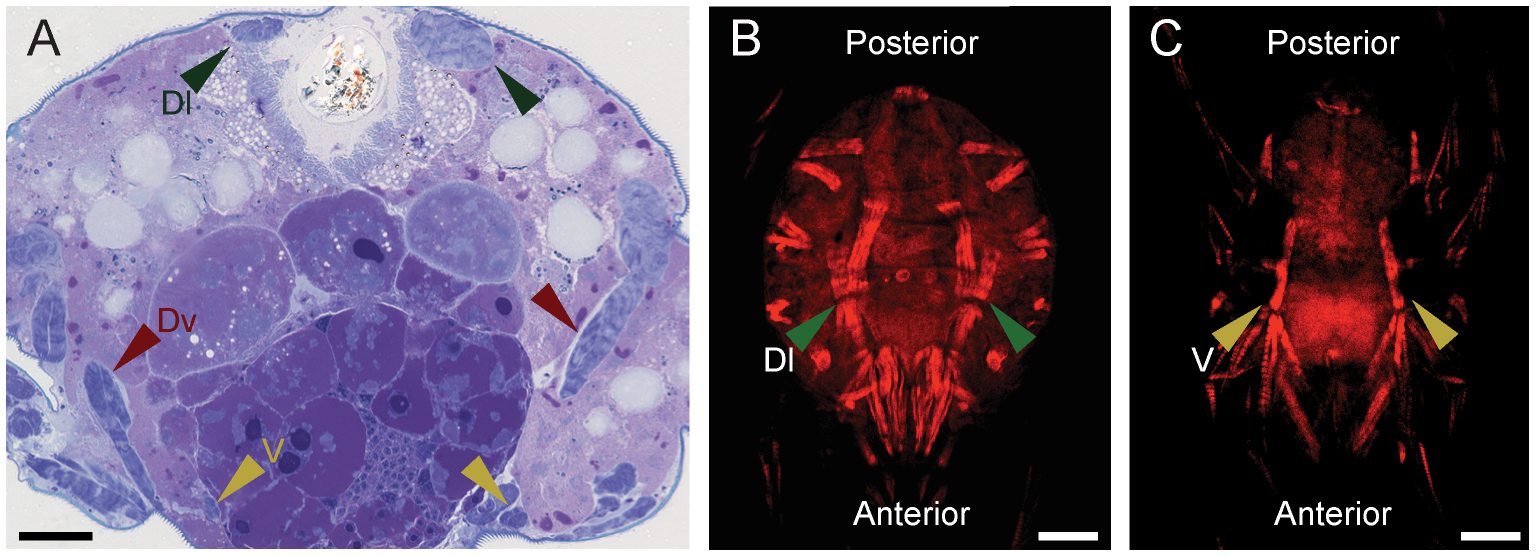

Supplement: Supplementary file 6 [file Image_5.tif]
